# Offene Verwaltungsdaten zur Wirkung bringen: Was sind die Schlüssel zum Erfolg?

**DOI:** 10.1365/s40702-021-00762-8

**Published:** 2021-08-02

**Authors:** Oliver Neumann, Tobias Mettler

**Affiliations:** Swiss Graduate School of Public Administration, University of Lausanne, Rue de la Mouline 28, 1022 Chavannes-près-Renens, Schweiz

**Keywords:** Offene Verwaltungsdaten, Digitale Transformation, Hackathons, Gemeinwohl, Öffentliche Verwaltung, Open government data, Digital transformation, Public value, Public administration

## Abstract

Die Digitalisierung schreitet auch im öffentlichen Sektor immer weiter voran. Ein wichtiger Aspekt der Verwaltungsdigitalisierung in vielen Ländern ist die Bereitstellung von Verwaltungsdaten als Open Government Data (OGD), auf Deutsch offene Verwaltungsdaten, denn öffentliche Organisationen produzieren und sammeln oft grosse Mengen an Daten und sind gleichzeitig zur Transparenz verpflichtet. Vermehrt stellt sich jedoch die Frage, wie offene Verwaltungsdaten auch über die Transparenz hinaus eine positive Wirkung im Sinne des Gemeinwohls leisten können und wie dies gefördert werden könnte. Im vorliegenden diskursiven Beitrag beschäftigen wir uns daher in drei Schritten mit den Voraussetzungen und Erfolgsfaktoren der Wirkungserzeugung durch offene Verwaltungsdaten. Erstens diskutieren wir, wie offene Verwaltungsdaten optimal publiziert werden können, so dass sie möglichst einfach von Dritten gefunden, gelesen und weiterbearbeitet werden können. Zweitens besprechen wir, was Wirkung in Bezug auf offene Verwaltungsdaten bedeutet, welche Arten von Wirkung unterschieden werden können und welche Mechanismen potenziell zu Wirkung führen. Drittens fokussieren wir noch spezifisch auf das Instrument der Innovationswettbewerbe, wie beispielsweise Hackathons, als möglichen Schlüssel zum Erfolg. Da öffentliche Organisationen sich vermehrt an solchen Veranstaltungen engagieren, geben wir einige konkrete Empfehlungen für die Vorbereitung und Teilnahme an diesen doch etwas besonderen Wettbewerben, bevor wir ein Fazit ziehen.

## Abstract (en)

The digital transformation in the public sector is steadily advancing. An important aspect in this process in many countries is the provision of administrative data as Open Government Data (OGD), since public organisations often produce and collect large amounts of data and are required to follow high standards of transparency at the same time. Increasingly, however, the question arises how OGD can have a positive impact on the common good beyond transparency and how this could be promoted. In this discursive article, we therefore look at the prerequisites and success factors for generating impact through OGD in three steps. First, we discuss how OGD can be optimally published so that they can easily be found, read and processed. Second, we discuss what impact means in relation to OGD, which types of impact can be distinguished and which mechanisms potentially lead to such effects. Third, we focus specifically on the instrument of innovation contests, such as hackathons, as a possible key to success. Since public organisations are participating more and more in such events, we provide some specific recommendations for preparing and participating in these somewhat special competitions before drawing a conclusion.

## Einleitung

Die Digitalisierung im öffentlichen Sektor zieht immer weitere Kreise. Eine wachsende Zahl öffentlicher Organisationen und Abteilungen arbeiten daran, ihre Prozesse und Dienstleistungen auf digitale Kanäle zu übertragen oder gar gänzlich neue, innovative Angebote basierend auf neuen Technologien zu schaffen. Vielerorts trägt zudem die Covid-19 Pandemie dazu bei, dass der Druck zur Transformation nochmals steigt und die Parole „weiter wie bisher“ immer seltener möglich ist beziehungsweise von den Bürgerinnen und Bürgern nicht mehr akzeptiert wird. Eines der derzeit häufigsten Phänomene der Verwaltungsdigitalisierung in vielen Weltregionen ist die systematische Bereitstellung von Verwaltungsdaten als *Open Government Data*, auf Deutsch *offene Verwaltungsdaten* (Wang und Lo 2016). Die Grundidee ist dabei, dass möglichst viele in der Verwaltung anfallende Daten frei, ohne Kosten und Zugangs- beziehungsweise Nutzungshürden sowie idealerweise in einem maschinenlesbaren Format zugänglich gemacht werden. Da öffentliche Organisationen oft mit zu den größten Produzenten beziehungsweise Sammlern von Daten in vielen verschiedenen Bereichen gehören, sind offene Verwaltungsdaten eine potenziell wertvolle Ressource für viele Anspruchsgruppen (Attard et al. 2016). Nicht umsonst werden Daten manchmal das Öl des 21. Jahrhunderts genannt – wobei bei genauerem Hinsehen Daten sogar Vorteile gegenüber Öl haben: sie können mehrfach verwendet werden, sind einfacher zu transportieren und können von Menschen und Maschinen nahezu unbegrenzt erzeugt werden.

Es gibt jedoch weiterhin grosse Diskussionen in Forschung und Praxis darüber, wie genau mit offenen Verwaltungsdaten ein tatsächlicher Mehrwert für die Allgemeinheit geschaffen werden kann und wie dieser Mehrwert genau aussieht. In einigen öffentlichen Verwaltungen, die bereits Daten publiziert haben, macht sich auch schon Ratlosigkeit oder gar Ernüchterung breit, weil die Nutzung der offenen Daten weit hinter den Erwartungen zurückbleibt und kaum relevante Ergebnisse entstehen. Während nämlich die politischen Strategien zu offenen Verwaltungsdaten häufig allerlei positive Effekte wie mehr Transparenz oder Innovationskraft als Grundannahme erwähnen, gehen sie oftmals in ihren Zielen kaum über die Förderung der Datenpublikation hinaus. Es fehlt also oft der Weitblick, um auch die proaktive Förderung der Datennutzung mit in die Strategie einzubeziehen und so auch dieses Feld zu bearbeiten. Denn wenn niemand die veröffentlichten Daten findet und niemand mit ihnen arbeitet, kann auch kein Nutzen daraus entstehen. Es gilt also Wege zu finden, wie Bürgerinnen und Bürger sowie weitere Akteure ausserhalb der Verwaltung dazu motiviert werden können, die Daten zu nutzen und die Ergebnisse zu einer möglichst breiten Anwendung zu bringen (Purwanto et al. 2020).

In diesem diskursiven Artikel beschäftigen wir uns daher mit den Voraussetzungen und Erfolgsfaktoren der Wirkungserzeugung durch offene Verwaltungsdaten. Im nächsten Abschnitt gehen wir zunächst darauf ein, wie offene Verwaltungsdaten optimal publiziert werden können. Zentral ist dabei, dass die Daten möglichst einfach von Dritten gefunden, gelesen und weiterbearbeitet werden können. Danach diskutieren wir, was Wirkung in Bezug auf offene Verwaltungsdaten bedeutet, welche Arten von Wirkung unterschieden werden können und welche Mechanismen potenziell zu Wirkung führen. Zuletzt werden wir dann noch konkreter und fokussieren spezifisch auf das Instrument der Innovationswettbewerbe als einen Schlüssel zum Erfolg. Von besonderer Relevanz in diesem Bereich waren in den letzten Jahren sogenannte Hackathons, auf denen immer mehr öffentliche Organisationen sich aktiv engagieren. Da solche Wettbewerbe jedoch für viele Verwaltungen sprichwörtlich noch Neuland sind, geben wir einige konkrete Empfehlungen für die Vorbereitung und Teilnahme an diesen doch etwas besonderen Wettbewerben, bevor wir ein Fazit ziehen.

## Offene Verwaltungsdaten optimal publizieren

Offene Verwaltungsdaten sollten mit Hinblick auf deren Nutzung publiziert werden (Francey und Mettler 2021). Anstatt eine undurchsichtige Flut unregelmäßig erscheinender und schlecht dokumentierter Datensätze zu produzieren, sollte der Fokus auf wenige, dafür hochwertige Datensätze gelegt werden. Dies auch, weil eine unzureichende Datenqualität ein ernsthaftes Risiko für den langfristigen Erfolg von Open Data Initiativen darstellt (Torchiano et al. 2017; Umbrich et al. 2015). Insbesondere wenn der Anspruch besteht, dass die öffentliche Verwaltung nicht nur Daten teilt, sondern die Daten durch Dritte auch genutzt werden sollen, ist es wichtig, dass die veröffentlichten Datensätze verständlich, vollständig, konsistent und maschinenlesbar sind (Janssen et al. 2012). Auch eine unklare Lizenzierung oder eine inkonsistente Preisgestaltung (speziell bei kommerzieller Nutzung) könnte eine spätere Verwendung durch Dritte beinträchtigen (Khayyat und Bannister 2015).

### Kriterien zur Bewertung der Qualität offener Verwaltungsdaten

Was „Qualität“ in Zusammenhang mit offenen Verwaltungsdaten genau bedeutet, wurde in zahlreichen Forschungsaufsätzen kontrovers diskutiert und in unterschiedlichen Qualitätsdimensionen komprimiert (vgl. Tab. 1). Grundsätzlich gilt es, mehrere Gesichtspunkte zu unterscheiden:

- die Beschaffenheit der Daten (resp. die Qualität der zur Verfügung gestellten Informationen),
- die Beschreibung der Daten und deren Nutzungsbedingungen (resp. die Qualität von Metadaten) sowie
- die Erschließung der Daten (resp. die Qualität der Open Data Portale und/oder Programmierschnittstellen).

**Tab. 1:** Kriterien zur Bewertung der Qualität offener Verwaltungsdaten aus Wissenschaft und Praxis (X = Kriterium wird in besagter Studie behandelt). Auf Grundlage von Marmier und Mettler (2020a)

*Unterschiedliche Kriterien zur Bewertung der Qualität offener Verwaltungsdaten*	(Vetrò et al. 2016)	(Máchová und Lnénicka 2017)	(Reiche und Höfig 2013)	(Umbrich et al. 2015)	(Reiche et al. 2014)	(Sunlight Foundation 2021)	(Open Knowledge Foundation 2021)	(Open Data Monitor 2021)	(European Data Portal 2021b)	(World Wide Web Foundation 2021)
Accessibility (Zugänglichkeit)	–	X	X	–	X	–	–	–	–	X
Accuracy (Fehlerfreiheit)	X	–	X	X	–	–	–	–	–	–
Availability (Verfügbarkeit)	–	X	X	–	–	–	–	X	X	–
Completeness (Vollständigkeit)	X	–	X	X	–	X	–	–	–	X
Compliance (Konformität)	X	–	–	–	–	–	–	–	–	–
Consistency (Konsistenz)	X	–	–	–	–	–	–	–	–	–
Easy access (Einfacher Zugang)	–	–	–	X	X	X	X	–	X	X
Free usage cost (Freie Nutzung)	–	–	–	–	–	X	X	–	X	X
License (Lizenzinformationen)	–	–	–	X	X	X	X	X	X	X
Machine readability (Maschinenlesbarkeit)	–	–	–	–	X	X	X	X	X	X
Metadata availability (Verfügbarkeit von Metadaten)	–	–	X	–	–	–	–	–	–	–
Metadata completeness (Vollständikeit von Metadaten)	–	–	X	–	–	–	–	X	–	–
Non-discrimination (Gleichbehandlung)	–	–	–	–	–	X	–	–	–	–
Open format (Offene Datenformate)	–	–	–	X	–	X	X	–	X	X
Permanence (Dauerhaftigkeit)	–	–	–	–	–	X	–	–	–	–
Primacy (Datenursprung)	–	–	–	–	–	X	–	–	–	–
Re-usability (Wiederverwendbarkeit)	–	–	X	X	–	–	–	–	X	–
Timeliness (Aktualität)	X	X	–	–	–	X	X	–	X	X

Viscusi et al. (2014) liefern ein Beispiel für eine Studie, die primär auf die Beschaffenheit der Daten zentriert ist. Auf Basis einer Analyse von 50 öffentlich zugänglichen Datensätzen bewerteten sie die Qualität der Daten in Bezug auf Vollständigkeit, Genauigkeit und Aktualität. Reiche et al. (2014) fokussieren sich hingegen auf die Analyse der veröffentlichten Metadaten auf Open Data Portalen, um Anhaltspunkte über die Qualität offener Verwaltungsdaten zu erhalten. Sie betrachteten dabei insbesondere, ob die Metadaten Aufschluss über die Maschinenlesbarkeit der Daten, deren Lizenzbestimmungen, sowie deren Zugänglichkeit geben. Umbrich et al. (2015) und Máchová und Lnénicka (2017) legen ihr Augenmerk auf das generelle Datenangebot und die Benutzerfreundlichkeit von Open Data Portalen. Während Erstere den Qualitätsbegriff als eine Kombination aus Wiederauffindbarkeit, Vollständigkeit, Genauigkeit, Offenheit und Erreichbarkeit definierten, konzentrierten sich Letztere auf Formalisierungsgrad, Vollständigkeit, Genauigkeit, Informationsreichtum, Zugänglichkeit und Verfügbarkeit. Beide Studien haben gemein, dass die Datensätze nur indirekt bewertet werden. Die Abschätzung der Qualität beruht primär auf Grundlage der Erschließung der Daten. Eine detaillierte Betrachtung der Beschaffenheit, Interpretierbarkeit und Nutzungsmöglichkeit der Daten respektive der eigentlichen Inhalte erfolgt also nicht.

Neben wissenschaftlichen Studien existieren auch zahlreiche weitere Bewertungshilfen, Leitfaden und Indizes, welche Aufschluss über die fortschreitende Verbreitung und Qualität von Open Data Initiativen geben. Ein Beispiel ist der *Global Open Data Index*, der eine Momentaufnahme des Datenangebots auf nationaler Ebene wiedergibt (Open Knowledge Foundation 2021). Weitere interessante Einblicke erhält man vom *Open Data Barometer* (World Wide Web Foundation 2021). Dieser zielt darauf ab, die Bereitschaft, die Implementierung sowie die Auswirkungen von Open Data Initiativen weltweit auf einer aggregierten Weise zu vergleichen. Der Barometer liefert zwar keine unmittelbaren Informationen über die Qualität der veröffentlichten Datensätze, veranschaulicht aber in einträglicher Manier, wo Betreiber nationaler Plattformen wesentlichen Nachholbedarf sehen. Auf ähnliche Weise präsentiert der *Open Data Monitor* unterschiedliche Indikatoren und Grafiken, welche den Reifegrad nationaler Open-Data-Bestrebungen innerhalb der Europäischen Union vergleichen sollen (European Data Portal 2021b). Informativ sind auch die Richtlinien der Sunlight Foundation (2021), welche versuchen, aufkommende „Good practices“ und bewährte „Best practices“ der Veröffentlichung von offenen Daten prägnant zusammenzufassen. Die in den Richtlinien empfohlenen Qualitätsdimensionen wurden von zahlreichen nationalen Open Daten Portalen (z. B. data.go.uk, data.gouv.fr, data.gov, etc.) als Grundlage für Benchmarks und für die Weiterentwicklung des Angebots herangezogen (Marmier und Mettler 2020a).

### Reifegradmodell für offene Verwaltungsdaten nach Berners-Lee (2021)

**Abb. 1:**
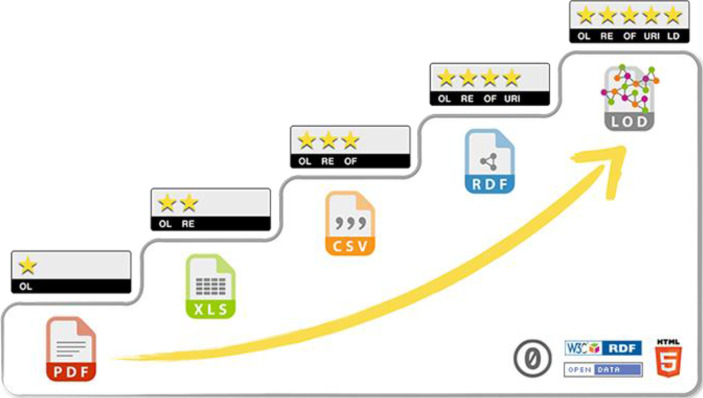
Reifegrade von Open Data: Publikation von PDF-Dokumenten bis hin zu Linked Open Data (Berners-Lee 2021, 2006)

Wie oben erwähnt, ist die Qualität der publizierten Daten maßgeblich für deren Nutzung. Doch wie steht es um die Wiederverwendbarkeit? Neben der reinen Information (z. B. verarbeitet in Form von Berichten oder integriert in Informationsbroschüren und dergleichen), können offene Verwaltungsdaten nämlich auch als Grundlage für neue Anwendungen (Apps), Datenbanken oder daten-basierter Visualisierungen dienen. Damit die von der öffentlichen Verwaltung publizierten Daten jedoch flexibel „wiederverwendet“ werden können, müssen diese in maschinenlesbarer Form und in nicht-proprietären Dateiformaten vorliegen. Tim Berners-Lee (2021), der „Vater des Webs“, hat unterschiedliche Reifegrade identifiziert, welche schrittweise Entwicklungsstufen beim Publizieren offener Verwaltungsdaten darstellen (vgl. Abb. 1).

Damit überhaupt von „offenen“ Verwaltungsdaten die Rede sein kann, müssen die publizierten Daten mittels einer entsprechenden Open-Data-Lizenz (*OL* *=* *open licence*) versehen worden sein. Diese schafft für Nutzerinnen und Nutzer eine gewisse Rechtssicherheit, indem eine ausdrückliche Erlaubnis erteilt wird, die Daten für nichtkommerzielle und/oder kommerzielle Zwecke nutzen zu können. Es existieren heute eine Vielzahl unterschiedlicher Lizenzmodelle. Um sich schnell einen Überblick zu verschaffen, hat das European Data Portal (2021a) einen Lizenz-Assistenten entwickelt, der bei der Auswahl und Deklaration behilflich sein kann.

Die zweite Entwicklungsstufe ergibt sich, wenn die Daten nicht nur als unstrukturierte Dokumente zur Verfügung gestellt werden (z. B. als PDF), sondern in eine maschinen-lesbare, das heisst strukturierte Form gebracht werden. Nur so können die veröffentlichten Daten wiederverwendet (*RE* *=* *re-usable*) werden. An dieser Stelle spielt es noch keine Rolle, welche Datenformate verwendet werden. Die Veröffentlichung von proprietären Formaten (z. B. Excel anstelle eines Bildscans einer Tabelle) wird hier gleichermaßen vorangetrieben, wie die von nicht-proprietären Formaten (z. B. CSV). Die ausschließliche Verwendung offener Dateiformate (*OF* *=* *open format*) wird nämlich erst in der dritten Entwicklungsstufe verlangt.

Um die Daten dauerhaft und eindeutig für die Nutzerinnen und Nutzer zugänglich zu machen, sollte in einer vierten Entwicklungsstufe darauf geachtet werden, einheitliche Bezeichner für Ressourcen (*URI* *=* *uniform resource identifier*) zu verwenden. Wird der Akzent auf die Wiederauffindbarkeit des Speicherortes (*location*) des Datensatzes gelegt, dann sollte der Datensatz mit einer eindeutiger URL versehen werden (was umgangssprachlich als Link bezeichnet wird). Möchte man einem Datensatz viel eher einen dauerhaft gültigen Namen (*name*) zuweisen, der den Datensatz eindeutig identifizierbar macht, kommt eine URN (*uniform resource name*) zum Zug. Wenn der Fokus nicht mehr nur auf die Verlinkung des eigenen Datensatzes, sondern auch auf die Herstellung von Kontext mit anderen Daten gelegt wird, dann hat man die letzte Entwicklungsstufe beim Publizieren offener Verwaltungsdaten erreicht (Hitz-Gamper et al. 2019).

## Mechanismen zur Wirkungserzeugung

Wenn öffentliche Organisationen beginnen, ihre Daten öffentlich zugänglich zu machen, stellt sich neben den technischen Fragen wie der optimalen Bereitstellung auch die Frage danach, wie aus den offenen Daten Wert geschaffen werden kann und wie dieser Wert aussehen kann. Die OECD geht davon aus, dass offene Verwaltungsdaten in vier Bereichen positive Auswirkungen auf die Gesellschaft haben können: Transparenz und Verantwortbarkeit von Politik und Verwaltung, Bürgerinklusion und -partizipation, Effektivität und Effizienz öffentlicher Organisationen sowie wirtschaftliches Wachstum (Davies 2013). Eine etwas andere, besonders in der Forschung verbreitete Perspektive ist die des *Public Value*, also der Erzeugung von Beiträgen an das Gemeinwohl durch offene Daten (Hitz-Gamper et al. 2019; Krishnamurthy und Awazu 2016; Pereira et al. 2017). Das Gemeinwohl ist dabei ein nicht vollständig klar abgrenzbarer Begriff, er beinhaltet jedoch nach Meynhardt (2009) eine moralisch-ethische, eine hedonistisch-ästhetische, eine politisch-soziale und eine utilitaristisch-instrumentelle Dimension. Unter der moralisch-ethischen Dimension sind Verbesserungen der Menschenwürde, Diversität, Integrität und Schutz der Privatsphäre gemeint, wohingegen die politisch-soziale Dimension Bürgerpartizipation, Chancengleichheit, Kompromissfähigkeit und soziale Innovation enthält. Die hedonistisch-ästhetische Dimension zielt auf den Schutz von Kulturerbe, die Förderung der Schönheit öffentlicher Orte, Zuverlässigkeit und Dienstleistungsqualität öffentlicher Organisationen ab, und die utilitaristisch-instrumentelle Dimension betrifft Förderung von Eigeninitiative, Offenheit und Transparenz, Robustheit und Nachhaltigkeit. Auch wenn all diese Dimensionen viel Spielraum für Interpretation lassen, so ist dennoch klar, dass Public Value beziehungsweise Gemeinwohl genau das ist, was den Kernauftrag öffentlicher Organisationen ausmacht und dementsprechend auch bei Initiativen im Bereich offener Daten im Zentrum stehen sollte.

**Abb. 2:**
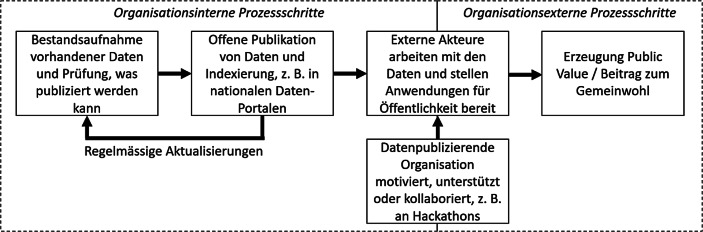
Ökosystem der Wertschöpfung durch offene Verwaltungsdaten

Oftmals wird jedoch übersehen, dass die genannten positiven Effekte kein unmittelbares Resultat davon sein können, dass Daten veröffentlicht werden (Löfgren und Webster 2020). Viele Organisationen machen den Fehler, das Thema offene Verwaltungsdaten als abgeschlossen zu betrachten, sobald die Daten online sind oder die neue Datenschnittstelle operativ ist. Die Perspektive bleibt also rein organisationsintern, das heisst es kommt oftmals zu einer Bestandsaufnahme vorhandener Daten und einer Prüfung, was publiziert werden kann (s. Kucera et al. 2015). Dabei können diverse politische, soziale, wirtschaftliche, organisationale, rechtliche und technische Hürden auftreten (Kucera et al. 2015), beispielsweise weil es Ängste bezüglich fehlerhafter Daten, rechtlicher Probleme oder unterschiedliche Vorstellungen darüber gibt, welche Daten für eine Publikation relevant genug sind. Eine Teilmenge der bei der Bestandsaufnahme erfassten Daten wird dann letztlich publiziert und gegebenenfalls regelmässig aktualisiert. Damit aus Daten tatsächlich ein Beitrag zum Gemeinwohl entstehen kann, ist es jedoch notwendig, dass externe Akteure Zeit und Ressourcen darin investieren, mit den Verwaltungsdaten zu arbeiten, beispielsweise um sie mit anderen Daten zu verlinken, sie zu visualisieren, zu analysieren oder zu interpretieren (Attard et al. 2016; Löfgren und Webster 2020). Zudem sollten die Ergebnisse idealerweise für die Öffentlichkeit auch zugänglich gemacht werden, so dass neue, auf Verwaltungsdaten basierende Applikationen von Interessierten auch genutzt werden können, oder aber privatwirtschaftliche Organisationen können ihre Geschäftsziele mit den Daten verfolgen, woraus positive wirtschaftliche Effekte entstehen können. Ein Beispiel für eine Firmengründung auf Basis von offenen Verwaltungsdaten ist das Startup Urbint in den USA, das basierend auf grossen Mengen offener Verwaltungsdaten und mittels künstlicher Intelligenz kommerzielle Risikoanalyse-Dienstleistungen für Projekte aller Art anbietet, beispielsweise für Infrastruktur‑, Kraftwerks- oder Gasförderungsprojekte. Die Aufgabe, Personen ausserhalb einer öffentlichen Organisation zur Arbeit mit den offenen Verwaltungsdaten zu stimulieren, ist also zentral und sollte von der datenanbietenden Organisation unbedingt ernst genommen werden (Susha et al. 2015). Es braucht also neben der organisationsinternen Perspektive, die auf die optimale Datenpublikation fokussiert, auch eine organisationsexterne Perspektive, die die möglichen Nutzerinnen und Nutzer der Daten in den Fokus nimmt (s. Abb. 2).

Aber wie genau können externe Personen zur Arbeit mit den offenen Daten motiviert werden? Diese Frage stellen sich viele öffentliche Organisationen nach der Datenpublikation und die Antwort kann vielschichtig sein. Laut einer Studie von Purwanto et al. (2020) gibt es einige grundlegende Faktoren, die zu mehr Bürgerengagement im Bereich offene Daten führen können. Dies sind unter anderem eine demokratische Kultur der Mitbestimmung in einer Gesellschaft, aber auch konkret beeinflussbare Faktoren wie ein möglichst einfacher Datenzugang, Unterstützungsangebote von öffentlichen Stellen bei Problemen, Datenqualität, Nutzung von sozialen Medien zur Kommunikation und soziale Beziehungen zwischen den beteiligten Gruppen. In Bezug auf den letzten Punkt ist insbesondere die Rolle von Communities, also mehr oder weniger losen Gruppen von an bestimmten Themenbereichen interessierten Bürgerinnen und Bürgern zu nennen. Aber öffentliche Organisationen sollten auch über ganz konkrete Veranstaltungen, Formate und Initiativen nachdenken (Marmier und Mettler 2020b), bei denen gezielt mit bestimmten Teilmengen der eigenen offenen Daten gearbeitet werden und so Wert erzeugt werden soll. Als hierfür besonders geeignetes Instrument haben sich in den letzten etwa zehn Jahren Innovationswettbewerbe herauskristallisiert (Johnson und Robinson 2014), über die wir im nächsten Abschnitt vertieft sprechen werden.

## Innovationswettbewerbe als Schlüssel zum Erfolg?

Öffentliche Organisationen sind es meist nicht gewohnt, ihre Problemstellungen öffentlich zu machen und auf diese Weise ausserhalb der Organisation beziehungsweise in der Zivilgesellschaft nach Lösungen zu suchen – ein Prinzip, dass als *Crowd-Sourcing* bezeichnet wird. Genau darum geht es jedoch bei Innovationswettbewerben wie Hackathons oder Makeathons (Almirall et al. 2014; Johnson und Robinson 2014; Sieber und Johnson 2015). Eine zentrale Arbeitsgrundlage sind dabei häufig offene Verwaltungsdaten. Die Relevanz solcher Wettbewerbe wird in der Schweiz auch von der aktuellen Strategie für offene Verwaltungsdaten auf Bundesebene (Schweizerische Eidgenossenschaft 2018) anerkannt. Sie sind also ein wichtiger werdendes und ausserhalb der Verwaltung bereits etabliertes Instrument, um Probleme mit der Hilfe von organisationsexternen Personen beziehungsweise Gruppen zu lösen. Die Eignung solcher Wettbewerbe zur Schaffung von Werten aus offenen Daten rührt daher, dass sie eine strukturierte Plattform für den direkten Austausch und Zusammenarbeit zwischen Datenanbieterinnen und Datenanbietern sowie interessierten Bürgerinnen und Bürgern sowie Firmen bieten (Johnson und Robinson 2014). So erhalten die datenanbietenden Organisationen die Möglichkeit, auf ihre Daten aufmerksam zu machen und zur Arbeit mit den Daten zu motivieren, aber auch auf direktem Wege Fragen zu beantworten, gemeinsame Ideen zu entwickeln oder auftretende Probleme zu lösen. Mit der Nutzung der zusätzlichen Ressourcen und Fähigkeiten der Teilnehmerinnen und Teilnehmer (die vielleicht innerhalb der Organisation nicht vorhanden sind) können Innovationswettbewerbe für öffentliche Organisationen also eine wichtige Antwort darauf sein, wie aus Daten Wert generiert werden kann.

Ein Beispiel für eine erfolgreiche Innovation, die auf offenen Daten einer öffentlichen Organisation entstanden in der Schweiz ist, kann die App SBB Inclusive der Schweizerischen Bundesbahnen genannt werden, mit welcher Blinde und seheingeschränkte Personen durch optische und akustische Informationen bei ihrer Reise mit öffentlichen Verkehrsmitteln unterstützt werden. Die App wurde an mehreren Innovationswettbewerben auf Basis offener Fahrplandaten und Daten zu Orientierungspunkten am Bahnhof sowie unter Beteiligung von Blindenverbänden entworfen und anschliessend organisationintern marktreif entwickelt, so dass sie heute produktiv genutzt werden kann. Als Beispiel aus Deutschland kann die App U:DO angeführt werden, die bei einem grossen Online-Wettbewerb namens WirVsVirus im Jahr 2020 entstand. Basierend auf frei zugänglichen Informationen zur Beantragung von Kurzarbeitergeld vereinfacht die Software den Beantragungsprozess substanziell und begleitet die Nutzerinnen und Nutzer über alle Schritte hinweg und verbessert so die Interaktionen zwischen Staat und Bürgerinnen und Bürgern.

**Abb. 3:**
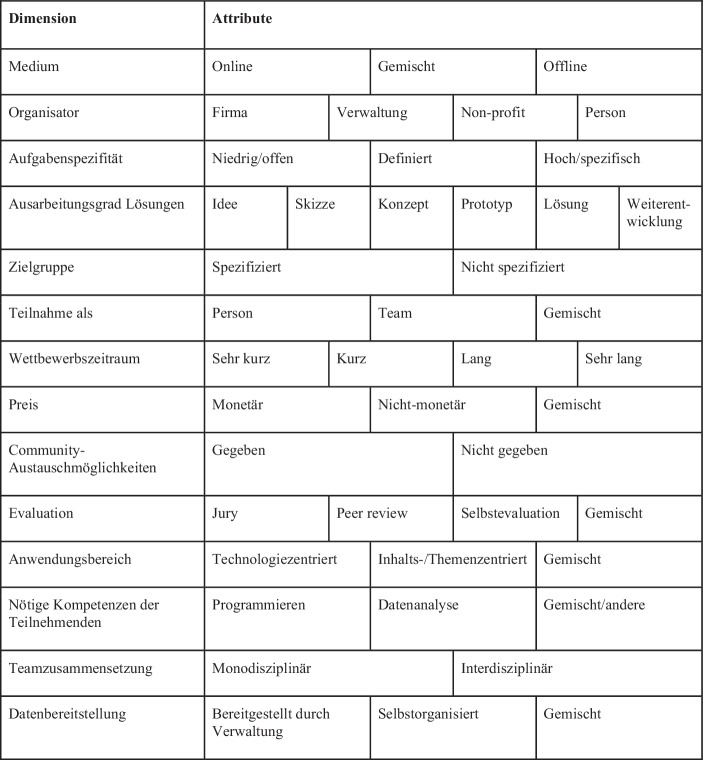
Ausgestaltungsmöglichkeiten für Open Data-bezogene Wettbewerbe, basierend auf Dinter und Kollwitz (2016)

Auch Dinter und Kollwitz (2016) befassen sich damit, wie und in welchen Bereichen Open Data im Rahmen von Innovationswettbewerben dafür eingesetzt werden kann, Innovationen in Zusammenarbeit mit Dritten voranzutreiben. Sie erachten dieses Instrument insbesondere für sinnvoll in Organisationen, die mit den eigenen Ressourcen und Fähigkeiten nicht in der Lage sind, ihre Daten für Innovationen zu nutzen. Es gibt jedoch verschiedene Formen von Innovationswettbewerben und nicht jede Form ist immer für jede Organisation geeignet. Dinter und Kollwitz (2016) haben deshalb für die Planung und Ausgestaltung solcher Innovationswettbewerbe verschiedene Dimensionen definiert, die jeweils mehrere Attribute beziehungsweise Ausgestaltungsoptionen enthalten. Abb. 3 enthält einen Auszug der relevantesten Dimensionen und dient Organisatorinnen und Organisatoren von Innovationswettbewerben mit Bezug zu Open Data dazu, sich aller relevanten Punkte während der Planung bewusst zu sein und sich gezielt für die Ausgestaltungsoptionen entscheiden zu können, die für den jeweiligen Zweck am dienlichsten sind. So kann beispielsweise bei der Dimension *Medium* entschieden werden zwischen einem Online-Format, einer Vor-Ort-Veranstaltung oder einer Mischung aus beidem. Bei der Dimension *Anwendungsbereich* kann entweder technologiezentriert oder eher themenzentriert gearbeitet werden – aber auch hier sind Mischungen möglich. Der *Wettbewerbszeitraum* kann von kurz bis lang dauern und auch die *Evaluation* der Ergebnisse des Wettbewerbs kann auf unterschiedliche Arten erfolgen, zum Beispiel durch eine Jury, durch Selbstevaluation, durch Peer-Review oder durch Mischbewertungen.

Nicht immer ist es vom Aufwand her jedoch empfehlenswert, als öffentliche Organisation einen eigenen Innovationswettbewerb auszurichten, denn die dafür notwendigen Ressourcen sind nicht zu unterschätzen. Das heisst aber nicht, dass man auf Innovationswettbewerbe verzichten muss, denn man kann auch als öffentliche Organisation eine sogenannte *Challenge*, also eine Herausforderung, bei einem bestehenden Wettbewerb einbringen. Eine häufig anzutreffende Form von Innovationswettbewerben im Zeitalter der Digitalisierung sind Hackathons (Almirall et al. 2014) – eine Wortkombination aus Hacking (nicht negativ gemeint, sondern als Programmieren zu verstehen) und Marathon. Hackathons dauern in der Regel 24 bis 72 h ohne Pause und finden oft vor Ort, manchmal aber auch online statt. Während dieser Zeit versuchen freiwillige Kreative, vorab definierte Herausforderungen der ebenfalls anwesenden datenanbietenden Organisationen zu lösen (Johnson und Robinson 2014). Oft gibt es für die besten Lösungen Preise zu gewinnen, aber meist arbeiten die Teilnehmerinnen und Teilnehmer vor allem aus Spass an der Lösungsentwicklung mit. Wichtig ist aus Sicht der Hackerinnen und Hacker, ein Team mit möglichst breiten Kompetenzen zusammenzustellen, das sowohl technische als auch fachliche Aspekte berücksichtigen kann (Almirall et al. 2014). Aber auch als veranstaltende beziehungsweise teilnehmende Organisation sollte man sich gemäss unserer eigenen Erfahrung Gedanken dazu machen, welches Publikum und damit welche Kompetenzen am Hackathon vertreten sind oder sein sollten, denn die Fähigkeiten der Anwesenden sollten zu den Challenges passen. In Europa und der Schweiz gibt es eine ganze Reihe sehr professioneller Hackathons. Einer der grössten Vor-Ort-Hackathons der letzten Jahre war jeweils der *HackZurich* mit über 500 internationalen Teilnehmerinnen und Teilnehmern. Im Bereich der Online-Hackathons sticht der oben bereits erwähnte *WirVsVirus*-Hackathon der Bundesregierung Deutschlands hervor, der im Pandemiejahr 2020 stattfand und sogar über 28.000 Teilnehmerinnen und Teilnehmer anzog. Aber auch bei kleineren Hackathons findet man oft eine professionelle Organisation und sehr motivierte Hackerinnen und Hacker.

Aus Sicht der datenbereitstellenden Organisation ist aus unserer Erfahrung bei einem Hackathon zudem vor allem eines wichtig: eine gute Vorbereitung, bei der eine spannende Herausforderung erarbeitet wird und für die die nötigen Daten bereits offen publiziert wurden. Denn wenn keine Hacker-Gruppe die mitgebrachte Herausforderung wirklich spannend findet, wird auch niemand sie wählen und bearbeiten – dann war die Vorbereitung umsonst. Berücksichtigt man einige Punkte, stehen die Chancen jedoch nicht schlecht: die Herausforderung sollte so gewählt sein, dass sie ein für Nutzerinnen und Nutzer relevantes Problem löst und keinesfalls auf ein rein organisationsinternes Problem abzielen. Die Hackerinnen und Hacker sollten sich möglichst mit der Herausforderung identifizieren können, ihre Lösung sollte einen möglichst direkten gesellschaftlichen Nutzen haben. Eine Challenge zum Thema Reduzierung der Bürokratie für Bürgerinnen und Bürger ist also gut, eine Challenge zur Verbesserung eines organisationinternen Controlling-Systems so gut wie chancenlos. Auch die richtige Flughöhe sollte getroffen werden. Die Challenge darf nicht zu einfach sein, aber auch nicht zu komplex, denn zwei oder drei Tage und Nächte sind kurz. Und man sollte sich auch über den Hackathon informieren, an dem man teilnimmt, denn teils unterscheiden sich die Hackathons in ihrer technischen und thematischen Ausrichtung. Behält man dann noch im Hinterkopf, dass bei einem Hackathon keine Lösungen entstehen können, die direkt produktiv eingesetzt werden können, sondern dass man dort maximal technische Ansätze, Ideen und Prototypen erhält, die man bei Gefallen selbst weiterentwickeln muss, ist man als öffentliche Organisation für eine Teilnahme gut gerüstet. Und nicht selten sind erstmalige Teilnehmerinnen beziehungsweise Teilnehmer an Hackathons recht schnell begeistert von der dort vorherrschenden Energie und Kreativität, auch wenn das intensive Durcharbeiten Tag und Nacht herausfordernd sein kann (natürlich ist ein paar Stunden schlafen aber erlaubt).

## Fazit

In diesem Beitrag haben wir uns mit der Frage beschäftigt, wie aus offenen Verwaltungsdaten ein Mehrwert entstehen kann. Hierfür ist es *organisationsintern* zunächst wichtig, die Qualität der Daten sicherzustellen und die Publikation möglichst professionell zu gestalten. Um dies in der Praxis zu erleichtern, haben wir eine Reihe von Kriterien zur Bewertung der Qualität offener Verwaltungsdaten sowie das Fünf-Sterne Reifegradmodell für offene Verwaltungsdaten nach Berners-Lee (2021) vorgestellt, welches Linked Open Data als höchsten Standard definiert. Ein wichtiges Prinzip ist es zudem, der Qualität der Daten gegenüber der Quantität tendenziell den Vorzug zu geben. Klar ist aber auch, dass Datensätze selten wirklich perfekt sind, und so muss man stets die richtige Balance zwischen Qualität und Quantität suchen. Kritisch betrachtet darf ein starker Fokus auf Qualität dann auch nicht bedeuten, die Quantität langfristig zu vernachlässigen oder mangelnde Qualität als Entschuldigung für eine nicht erfolgte Datenpublikation zu betrachten. Denn im Grunde genommen gibt es kaum Argumente, warum öffentliche Organisationen langfristig nicht alle ihre nicht besonders geschützten Daten im Sinne der Transparenz veröffentlichen sollten. Hierbei spielt jedoch auch eine politische Komponente eine Rolle und es liegt an den politischen Verantwortlichen, das Prinzip „offene Daten als Standard“ zu definieren.

Ebenfalls einen sehr hohen Stellenwert sollte aber auch die *organisationsexterne* Perspektive haben: Es gilt, externe Personen zur Arbeit mit den eigenen Daten zu motivieren, sie zu unterstützen und mit ihnen zusammenzuarbeiten. Dies bietet die Chance, als öffentliche Organisation oder Verwaltungen von den Kompetenzen und Fähigkeiten externer Personen zu profitieren so neue Ideen und Lösungsansätze zu generieren. Im Idealfall entstehen so neue Lösungen, die Mehrwerte für das Gemeinwohl schaffen – unter der Voraussetzung, dass die Datenqualität gut ist (s. oben). Längerfristig können sogar Communities, wie beispielsweise in der Schweiz der Verein OpenData.ch oder in Deutschland die Initiative #UpdateDeutschland, entstehen, die sich über einen längeren Zeitraum mit den Daten und Herausforderungen des öffentlichen Sektors auseinandersetzen und so die Fähigkeit zur Problemlösung öffentlicher Organisationen verbessern können. Ein zunehmend populärer Weg, Werte aus Verwaltungsdaten zu schaffen, sind Innovationswettbewerbe wie Hackathons. Solche Formate können zudem eine Alternative zu starren Beschaffungsverfahren sein, wenn es darum geht, schnell neue Lösungen zu entwickeln, und sie fördern das zivilgesellschaftliche Engagement für den öffentlichen Sektor (Johnson und Robinson 2014). Auch wenn nicht immer bei jedem Hackathon unmittelbar verwertbare Resultate entstehen, und auch wenn selbst die besten Resultate noch viel weiterer, organisationsinterner Arbeit bedürfen, bis eine Lösung produktiv einsetzbar ist, so zeigen Erfolgsbeispiele wie SBB Inclusive, U:DO aus dem WirVsVirus Hackathon oder das kommerzielle Startup Urbint in den USA dennoch, dass Hackathons für öffentliche Organisationen durchaus ein Schlüssel zum Erfolg bei der Schaffung von Werten aus ihren Daten sein können. Wichtig ist dabei, dass Hackathons nicht nur als Marketinginstrument verstanden werden, sondern auch tatsächlich die organisationsinternen Fachexpertinnen und Fachexperten teilnehmen und mitarbeiten. Wir haben darum diskutiert, welche Faktoren öffentliche Organisationen beachten sollten, wenn sie an Innovationswettbewerben mit ihren offenen Daten und ihren Herausforderungen teilnehmen (oder gar selbst solche Wettbewerbe ausrichten) möchten. Insbesondere gilt es dann, den passenden Hackathon auszuwählen, eine spannende Challenge zu formulieren und dabei rein organisationsinterne Problemstellungen ohne Bezug zu Bürgerinnen und Bürgern zu vermeiden. Zudem muss die Bereitschaft da sein, auch nach dem Hackathon weiter an der Lösung zu arbeiten und sie zu perfektionieren. Wir erwarten, dass Hackathons weiter an Wichtigkeit gewinnen werden und regen öffentliche Organisationen dazu an, sich auf dieses zunächst sicherlich ungewöhnliche Abenteuer einzulassen.

## References

[CR1] Almirall E, Lee M, Majchrzak A (2014) Open innovation requires integrated competition-community ecosystems: lessons learned from civic open innovation. Bus Horiz 57(3):391–400. 10.1016/j.bushor.2013.12.009

[CR2] Attard J, Orlandi F, Auer S (2016) Value creation on open government data. In: Proceedings of the 49th Hawaii international conference on system sciences 10.1109/HICSS.2016.326

[CR3] Berners-Lee T (2006) Linked data-design Issues. http://www.w3.org/DesignIssues/LinkedData.html. Zugegriffen: 13. März 2021

[CR4] Berners-Lee T (2021) 5 ★ Open data. https://5stardata.info/en. Zugegriffen: 13. März 2021

[CR5] Davies T (2013) Open data barometer – 2013 global report. http://www.opendataresearch.org/dl/odb2013/Open-Data-Barometer-2013-Global-Report.pdf. Zugegriffen: 13. März 2021

[CR6] Dinter B, Kollwitz C (2016) Towards a framework for open data related innovation contests. Proceedings of the Pre-ICIS SIGDSA/IFIP WG. 3 Symposium.

[CR7] European Data Portal (2021a) Lizenz-Assistent. https://www.europeandataportal.eu/de/training/licensing-assistant. Zugegriffen: 13. März 2021

[CR8] European Data Portal (2021b) Open data in Europe. https://www.europeandataportal.eu/en/dashboard/2020. Zugegriffen: 13. März 2021

[CR9] Francey A, Mettler T (2021) The effects of open government data: some stylised facts. Inf Polity. 10.3233/IP-200281

[CR10] Hitz-Gamper BS, Neumann O, Stürmer M (2019) Balancing control, usability and visibility of linked open government data to create public value. Int J Public Sector Manag 32(5):451–466. 10.1108/IJPSM-02-2018-0062

[CR11] Janssen M, Charalabidis Y, Zuiderwijk A (2012) Benefits, adoption barriers and myths of open data and open government. Inf Syst Manag 29(4):258–268. 10.1080/10580530.2012.716740

[CR12] Johnson P, Robinson P (2014) Civic hackathons: Innovation, procurement, or civic engagement? Rev Policy Res 31(4):349–357. 10.1111/ropr.12074

[CR13] Khayyat M, Bannister F (2015) Open data licensing: more than meets the eye. Inf Polity 20(4):231–252. 10.3233/IP-150357

[CR14] Krishnamurthy R, Awazu Y (2016) Liberating data for public value: the case of data.gov. Int J Inf Manage 36(4):668–672. 10.1016/j.ijinfomgt.2016.03.002

[CR15] Kucera J, Chlapek D, Klímek J, Necaský M (2015) Methodologies and best practices for open data publication. DATESO 2015, S 52–64

[CR16] Löfgren K, Webster CWR (2020) The value of big data in government: the case of ‘smart cities. Big Data Soc 7(1):2053951720912775. 10.1016/j.ijinfomgt.2016.05.002

[CR18] Marmier A, Mettler T (2020a) Developing an index for measuring OGD publisher compliance to good practice standards: Insights from opendata. swiss. Inf Polity 25(1):91–110. 10.3233/IP-180120

[CR19] Marmier A, Mettler T (2020b) Different shades of perception: how do public managers comprehend the re-use potential of open government data? Proceedings of the 41st International Conference on Information Systems.

[CR20] Meynhardt T (2009) Public value inside: what is public value creation? Int J Public Adm 32(3–4):192–219. 10.1080/01900690902732632

[CR17] Máchová R, Lnénicka M (2017) Evaluating the quality of open data portals on the national level. J Theor Appl Electron Commer Res 12(1):21–41. 10.4067/S0718-18762017000100003

[CR21] Open Data Monitor (2021) The open data monitor. https://opendatamonitor.eu/. Zugegriffen: 13. März 2021

[CR22] Open Knowledge Foundation (2021) Tracking the state of open government data. https://index.okfn.org/place/. Zugegriffen: 13. März 2021

[CR23] Pereira GV, Macadar MA, Luciano EM, Testa MG (2017) Delivering public value through open government data initiatives in a Smart City context. Inf Syst Front 19(2):213–229

[CR24] Purwanto A, Zuiderwijk A, Janssen M (2020) Citizen engagement with open government data. Transform Gov People Proc Policy 14(1):1–30. 10.1108/TG-06-2019-0051

[CR25] Reiche KJ, Höfig E (2013) Implementation of metadata quality metrics and application on public government data. Proceedings of the 37th Annual Computer Software and Applications Conference Workshops. 10.1109/COMPSACW.2013.32

[CR555] Reiche KJ, Höfig E, Schieferdecker I (2014) Assessment and visualization of metadata quality for open government data. Proceedings of the 4th Conference for E-Democracy and Open Government

[CR26] Schweizerische Eidgenossenschaft (2018) Strategie für offene Verwaltungsdaten in der Schweiz 2019–2023. https://www.fedlex.admin.ch/eli/fga/2019/125/de. Zugegriffen: 13. März 2021

[CR27] Sieber RE, Johnson PA (2015) Civic open data at a crossroads: dominant models and current challenges. Gov Inf Q 32(3):308–315. 10.1016/j.giq.2015.05.003

[CR28] Sunlight Foundation (2021) Ten principles for opening up government information. https://sunlightfoundation.com/policy/documents/ten-open-data-principles/. Zugegriffen: 13. März 2021

[CR29] Susha I, Grönlund Å, Janssen M (2015) Organizational measures to stimulate user engagement with open data. Transform Gov People Proc Policy 9(2):181–206. 10.1108/TG-05-2014-0016

[CR30] Torchiano M, Vetrò A, Iuliano F (2017) Preserving the benefits of open government data by measuring and improving their quality: an empirical study. Proceedings of the 41st Annual Computer Software and Applications Conference. 10.1109/compsac.2017.192

[CR31] Umbrich J, Neumaier S, Polleres A (2015) Quality assessment and evolution of open data portals. Proceedings of the 3rd International Conference Future Internet of Things and Cloud. 10.1109/FiCloud.2015.82

[CR32] Vetrò A, Canova L, Torchiano M, Minotas CO, Iemma R, Morando F (2016) Open data quality measurement framework: definition and application to open government data. Gov Inf Q 33(2):325–337. 10.1016/j.giq.2016.02.001

[CR33] Viscusi G, Spahiu B, Maurino A, Batini C (2014) Compliance with open government data policies: an empirical assessment of Italian local public administrations. Inf Polity 19(3/4):263–275. 10.3233/IP-140338

[CR34] Wang H‑J, Lo J (2016) Adoption of open government data among government agencies. Gov Inf Q 33(1):80–88. 10.1016/j.giq.2015.11.004

[CR35] World Wide Web Foundation (2021) Open data barometer. https://opendatabarometer.org. Zugegriffen: 13. März 2021

